# Facile Electrochemical Sensor for Sensitive and Selective Determination of Guaifenesin, Phenylephrine and Paracetamol on Electrochemically Pretreated Pencil Graphite Electrode

**DOI:** 10.3390/mi13081213

**Published:** 2022-07-29

**Authors:** Mihaela Buleandră, Anca Aurelia Pătrașcu, Dana Elena Popa, Iulia Gabriela David, Irinel Adriana Badea, Anton Alexandru Ciucu

**Affiliations:** Department of Analytical Chemistry, Faculty of Chemistry, University of Bucharest, Panduri Av. 90-92, District 5, 050663 Bucharest, Romania; patrascu.anca16@gmail.com (A.A.P.); elena.popa@chimie.unibuc.ro (D.E.P.); irinel.badea@chimie.unibuc.ro (I.A.B.); anton.ciucu@chimie.unibuc.ro (A.A.C.)

**Keywords:** guaifenesin, phenylephrine, paracetamol, pencil graphite electrode, differential pulse voltammetry, pharmaceutical samples

## Abstract

Guaifenesin (GFS), phenylephrine (PHE) and paracetamol (PAR), drugs used in combination for the relief of cold and flu symptoms, were determined at electrochemically pretreated pencil graphite electrode. Differential pulse voltammetry (DPV) was used for the first time for the concomitant determination of the target compounds based on the electro-oxidation of PAR at 0.43 V, PHE at 0.74 V and GFS at 1.14 V in Britton–Robinson buffer pH 6.0. Under optimized experimental conditions, two linear ranges were obtained for PAR (2.50 × 10^−6^ M–1.00 × 10^−5^ M and 1.00 × 10^−5^ M–1.00 × 10^−4^ M) and for PHE and GFS linearity was proved between 5.00 × 10^−6^ M–2.00 × 10^−4^ M and 2.50 × 10^−6^ M–2.00 × 10^−4^ M, respectively. The detection limits were 8.12 × 10^−7^ M for PAR, 1.80 × 10^−6^ M for PHE and 8.29 × 10^−7^ M for GFS. The selective and sensitive DPV method and the electrochemically treated electrode were employed for simultaneous analysis of the analytes in pharmaceutical samples with good recoveries.

## 1. Introduction

Guaifenesin (GFS), phenylephrine (PHE) and paracetamol (PAR) are active ingredients frequently combined in pharmaceutical formulations administered for short-term treatment of cold and flu symptoms (pain, nasal congestion, headache, fever and chesty cough) [1]. In such over-the-counter drugs, guaifenesin (3-(2-methoxyphenoxy)-1,2-propanediol) is an expectorant [2], phenylephrine (2-methylamino-1-3(-hydroxyphenyl)ethanol) is used as a decongestant [3] and paracetamol (N-(4-hydroxyphenyl) acetamide) acts as an analgesic and antipyretic agent [4].

The quality control of such preparations is of special importance considering that the overdoses can cause a range of hepatic damages (PAR) [5], depression of the central nervous system (GFS) [6] or severe hypertension and tachycardia (PHE) [7]. Therefore, the rigorous quantitative determination of these chemical compounds is of great interest. However, a challenge in the analysis of pharmaceutical preparations consists in the simultaneous detection, without preliminary separation, of more active ingredients with similar physico-chemical properties, from the complex formulations that contain a wide range of excipients. 

Being probably the most used drug worldwide, PAR has been widely studied, as evidenced by the many analytical methods developed for its quantification [8,9,10]. Moreover, there are some analytical methods used for the determination of GFS, PHE and PAR combined with other active ingredients in various pharmaceutical products. Many of these are based on chromatographic [11,12] and spectrophotometric techniques [13,14,15]. Due to the recognized advantages, electrochemical methods are a valuable alternative to overcome the drawbacks of other analytical methods which are expensive and time consuming. 

Despite the fact that there are many published papers on the individual electrochemical determination of the three mentioned drugs, but also in combination with other active substances, the literature survey revealed that there is no reported electrochemical method for the simultaneous analysis of the ternary mixture of GFS, PHE and PAR in pharmaceutical formulations. Thus, PAR was determined together with GFS in the presence of ascorbic acid [16] or oxomemazine hydrochloride [17] using modified carbon paste electrodes. There are more electrochemical methods proposed for the determination of PAR and PHE, these active ingredients being quantified in their binary mixtures [18,19] or together with chlorpheniramine maleate [20], dextromethorphan [21], cetirizine [22], ascorbic acid [23] or loratadine [24]. In all these studies chemically modified electrodes based on carbon paste or glassy carbon, but also boron-doped diamond electrodes were used.

A cheaper and simple alternative to these modified electrodes that require expensive reagents and additional preparation steps is the pencil graphite electrode (PGE). Further, the low cost and disposable use that eliminates the tedious cleaning procedures, PGE benefits from the excellent properties of composite graphite [25,26]. PGE was used as PAR electrochemical sensor in pharmaceutical formulations and different biological samples, the literature data on this subject being presented in a review paper [27]. For GFS determination, PGE was modified with silver nanoparticles and poly (L-cysteine) [28], but for PHE quantification there is no study that uses this type of electrode. 

Therefore, the main objective of the present study was to develop a differential pulse voltammetric (DPV) method which was able to quickly and selectively determine, in a single anodic scan, GFS, PHE and PAR from pharmaceutical formulations using the electrochemically pretreated PGE (PGE*). Moreover, the electrochemical behavior of all pharmaceutical active ingredients was studied at the PGE* surface.

## 2. Materials and Methods

### 2.1. Reagents and Apparatus

The stock standard solutions of 1.00 × 10^−2^ M PAR, PHE and GFS were daily prepared by dissolving the corresponding reagent purchased from Merck in double distilled water and were stored in the refrigerator until further use. The chemicals needed to obtain Britton–Robinson (BR) supporting electrolyte solutions (acetic acid, phosphoric acid, boric acid and sodium hydroxide) were also acquired from Merck. 

Sachets with powder for oral solution containing paracetamol (500 mg), guaifenesin (200 mg) and phenylephrine hydrochloride (10 mg) were bought from a local pharmacy. 

Cyclic voltammetry (CV) and DPV were performed using an analytical system model Autolab PGSTAT 128 N controlled by Nova 1.11 software (Ecochemie B.V., Netherlands). A glass cell containing 10 mL of solution and a three electrode system were used: PGE* as working electrode (if not stated otherwise), Ag/AgCl (3.00 M KCl) and platinum wire as reference and counter electrodes, respectively. Rotring graphite pencil leads with different levels of hardness (2H, H, HB, B and 2B) and diameter of 0.50 mm constituted the working electrode. The length of the lead inserted into the solution was 1.00 cm, PGE being prepared according to our previous works [26,29].

All pH measurements were carried out with a Consort C6010 pH/mV-meter (Fisher Scientific, Merelbeke, Belgium) at room temperature. 

### 2.2. Electrochemical Pretreatment of PGE

The PGEs were electrochemically activated using an already described procedure [30]. Thus, ten voltammetric cycles between −0.20 V and 3.00 V at a scan rate of 0.50 V/s were performed in a BR buffer solution pH 2.21. Moreover, surface characterization of PGE and PGE* (HB type) was performed by atomic force microscopy [29] and by CV [31].

### 2.3. Sample Analysis

Three sachets containing the pharmaceutical mixture were examined, five replicate samples being analyzed from each sachet. The content of one sachet with powder for oral solution was prepared according to the label instructions: it was dissolved in 250 mL warm water and then allowed to cool to room temperature. The thus obtained solution was further diluted with the appropriate supporting electrolyte such that the concentration of the sample subjected to the voltammetric measurement fell within the linear range.

For the quantitative determination of GFS, PHE and PAR the standard addition method was applied. Thereby, three different volumes of the stock standard solution were added into the volumetric flasks containing the same diluted sample volume, each time the final concentration falling into the linear range. Taking into consideration that the declared contents of the target compounds in the pharmaceutical formulation were significantly different, the standard addition method was performed for each analyte at a time.

Differential pulse voltammograms were recorded for the diluted sample solution and for each of the solutions obtained after the additions were made.

## 3. Results and Discussion

### 3.1. Electrochemical Behavior of PAR, PHE and GFS at PGE*

**Figure 1:**
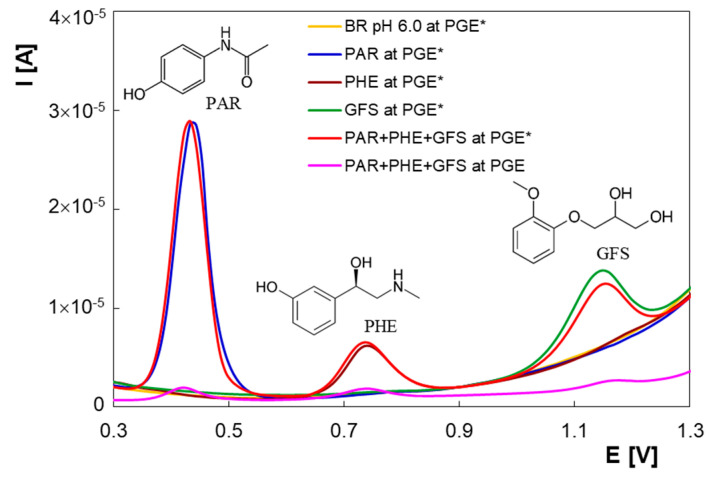
Differential pulse voltammograms of 2.00 × 10^−5^ M PAR, 2.00 × 10^−5^ M PHE, 2.00 × 10^−5^ M GFS and a mixture of PAR, PHE and GFS (2.00 × 10^−5^ M each) at PGE and PGE* in BR buffer pH 6.00.

It is well-known that simultaneous determination of electroactive compounds is sometimes difficult due to their voltammetric responses overlapping. In the present study DPV measurements were realized in order to evaluate the electrochemical responses of PAR, PHE and GFS in BR buffer solution pH 6.00 at PGE and PGE*, respectively. In order to verify the possibility of simultaneous determination of the three compounds, electrochemical experiments were firstly performed for each analyte (Figure 1). Thus, at PGE*, in the solution containing PAR a well-defined anodic voltammetric response was obtained at 0.43 V. For PHE, the electrochemical signal was at 0.74 V, while GFS presented an anodic peak at 1.14 V. In the differential pulse voltammogram recorded for the drugs mixture solution distinct electrochemical signals were observed at the same potentials as in the individual voltammograms, which can be attributed to the oxidation of PAR, PHE and GFS, respectively. The significant differences between the peak potentials of the analytes (0.31 V and 0.40 V for PAR-PHE and PHE-GFS, respectively) made possible the simultaneous determination of PAR, PHE and GFS in their mixture solution. 

Moreover, a comparison of differential pulse voltammograms registered at PGE and PGE* revealed considerably increased faradaic currents of PAR, PHE and GFS at the electrochemically pretreated electrode surface (Figure 1). During the electrochemical activation the PGE surface becomes oxidized and more hydrophilic, and the increasing number of oxygen-containing functionalities (hydroxyl, carboxyl, epoxide, etc.) along with surface edge-plane sites are responsible for the improved electron transfer at the interface electrode–electrolyte [26,30]. This behavior was translated into greatly improved oxidation signals for PAR, PHE and GFS. These results proved that the sensitivity of the simultaneous determination of PAR, PHE and GFS was enhanced at the PGE* surface. 

The pencil graphite is a composite material with different hardness due to the different ratio of graphite, clay and binder, their composition affecting the electrochemical properties of PGE [25]. For this reason, the DPV oxidation of PAR, PHE and GFS was investigated in BR buffer solution pH 6.00 with electrochemically pretreated pencil leads of different hardness (2B, B, HB, H and 2H). The sensor sensitivity S, expressed as A × M^−^^1^ × cm^−^^2^, for all five types of graphite leads was calculated and compared in order to establish which electrode is the most effective. The results were: 0.40 (HB) > 0.14 (H) > 0.10 (2B) > 0.08 (2H) > 0.06 (B) for PAR, 0.29 (HB) > 0.17 (H) > 0.16 (2B) > 0.15 (2H) > 0.12 (B) for PHE and 0.14 (HB) > 0.12 (2H) = 0.12 (2B) > 0.11 (B) > 0.09 (H) for GFS. These results emphasized that the most sensitive signals were obtained at HB leads, this kind of lead being used as electrode material in the next experiments. 

**Figure 2:**
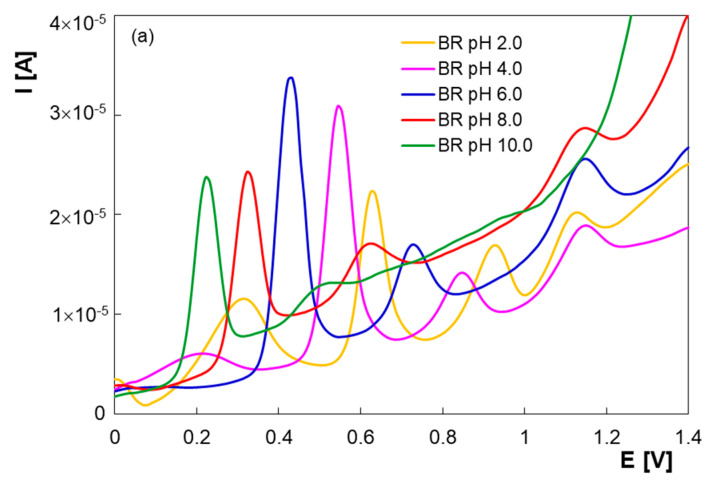

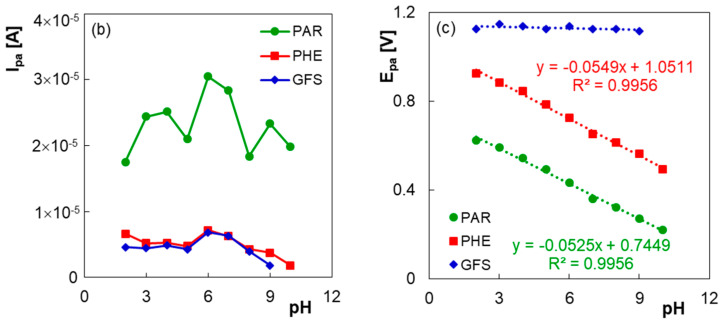
Differential pulse voltammograms (selection) of a mixture of 2.00 × 10^−5^ M PAR, 2.00 × 10^−5^ M PHE and 2.00 × 10^−5^ M GFS at PGE* in BR buffer solutions with different pH values (**a**); graphical representation of I_pa_ vs. pH (**b**); graphical representation of E_pa_ vs. pH (**c**).

The pH of the supporting electrolyte has a significant influence on the electrochemical measurements. In this context, in order to obtain the best resolution and the maximum sensitivity, DPV studies were performed in BR buffer solutions in the pH range 2.00–10.00 for ternary mixtures of PAR, PHE and GFS (Figure 2a). At pH values between 2.00 and 4.00, besides the three signals assigned to the target analytes, a broad signal at lower potentials was observed, which decreased with pH increasing and was probably due to the supporting electrolyte. Furthermore, at pH 2.00 the difference between the peak potentials of PHE and GFS was 0.20 V, the peak separation being more obvious at higher pH values. Figure 2b shows that the peak currents (I_pa_) were maximum at pH 6.00.

The anodic peak potentials (E_pa_) of PAR and PHE were shifted towards less positive potentials with pH increasing (Figure 2c), suggesting protons involvement in the electrochemical processes. The behavior of GFS was significantly different, the peak potential shifting being only 0.004 V per unit of pH. GFS peak potential remained almost constant, while the one of PHE decreased and, in this way, the separation between the GFS and PHE peaks increased considerably. The slopes of −0.0525 V/pH and −0.0549 V/pH for PAR and PHE (Figure 2c) suggested an equal number of protons and electrons transferred in the oxidation reactions. On the other hand, GFS oxidation peak potential was almost the same for different pH values, indicating the unavailability of protons that promote the GFS oxidation. All its functional groups are alcohols, which are very weak acids and so none of them are involved in proton transfer equilibrium at a pH lower than 13.62 (the pK_a_ of GFS). Thus, GFS is not a proton donor and therefore its oxidation is not pH dependent between 0 and 13.62. 

Considering the results of the pH dependence study, BR buffer solution with pH 6.00 was selected for PAR, PHE and GFS simultaneously detection in the subsequent experiments. 

**Figure 3:**
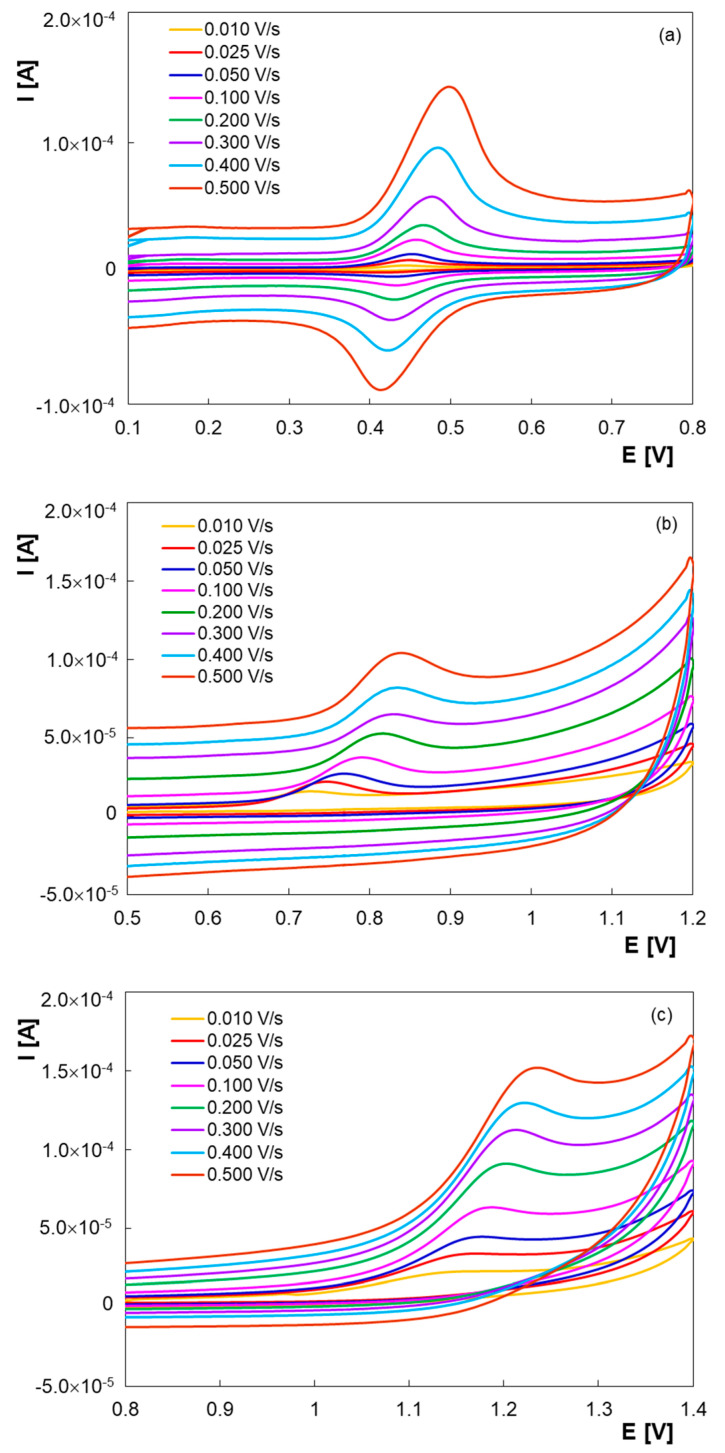
Cyclic voltammograms of 1.00 × 10^−4^ M PAR (**a**), 1.00 × 10^−4^ M PHE (**b**) and 1.00 × 10^−4^ M GFS (**c**) in BR buffer solutions pH 6.00 at PGE* at different scan rate values.

In order to obtain more information about the electrode processes, the scan rate effect on the peak currents and potentials of 1.00 × 10^−4^ M PAR (Figure 3a), 1.00 × 10^−4^ M PHE (Figure 3b) and 1.00 × 10^−4^ M GFS (Figure 3c) in BR buffer solution pH 6.00 was investigated in the range of 0.01 to 0.50 V/s by CV. Cyclic voltammograms for PAR (I_pa_/I_pc_ > 1, the peak potentials shifted with the scan rate increase) revealed a quasireversible behavior at PGE* surface, while no cathodic peak was observed at the reverse scan for PHE and GFS, which indicated that the charge transfer during the electrode process was completely irreversible.

The log of peak currents for all three analytes were linearly proportional to the log of the scan rate (v): log I_pa_PAR_ = 0.9420 log v—3.7972 (R^2^ = 0.9989), log I_pc_PAR_ = 1.0396 log v—4.0428 (R^2^ = 0.9985), log I_pa_PHE_ = 0.5165 log v—4.4911 (R^2^ = 0.9977) and log I_pa_GFS_ = 0.5435 log v—4.1636 (R^2^ = 0.9995). The slopes of the regression equations indicated that the electro-oxidation of PHE and GFS followed diffusion-controlled electron transfers at PGE*, for PAR being confirmed an adsorption controlled process. 

According to Laviron’s theory [32], the slopes of the plots of E_pa_ and E_pc_ vs. log v are equal to 2.3RT/(1 − α)nF and −2.3RT/αnF for the oxidation and reduction peaks, respectively and can be used to estimate the electron number (n) transferred in the electrochemical process and the transfer coefficient (α). At scan rates higher than 0.20 V/s the oxidation and reduction peak potentials of PAR were linear with log of the scan rate: E_pa_ [V] = 0.0736 log v + 0.5112 (R^2^ = 0.9958) and E_pc_ [V] = –0.0747 log v + 0.3893 (R^2^ = 0.9947). Based on this information, the transfer coefficient and the electron number were determined to be 0.41 and 2, respectively. 

The mechanism of the electrochemical oxidation of PAR was extensively studied and the literature data confirm our results [33,34]. Thus, PAR electro-oxidation involves the transfer of two electrons and two protons, generating *N*-acetyl-*p*-benzoquinone-imine that participates in dimerization, hydroxylation or hydrolyzes reactions depending on the solution’s pH [35]. 

The electron number transferred in the electrochemical oxidation of PHE and GFS was calculated using the relation E_pa_ – E_p/2_ (V) = 0.047/(1 − α)n [36], where E_p/2_ is the potential at which the current is at half maximum in the cyclic voltammogram. Taking into consideration that α = 0.5 for an irreversible system and the calculated values for (1 − α)n were 0.558 and 1.009, n was estimated as 1 for PHE and 2 for GFS. 

Thus, the reaction mechanism of PHE on PGE* involved one electron and one proton. In accordance with the existing studies PHE oxidation takes place at the phenolic group [37] and may lead to a phenoxyl radical which will further polymerize [38]. 

The number of electrons estimated to be involved in GFS electro-oxidation at PGE* is similar to the data reported in the literature [39,40]. One can suppose that under the electrochemical conditions described above, two electrons are needed for GFS’s diol moiety to undergo a mild oxidation leading to a α-hydroxy ketone [41]. However, more detailed investigations are necessary in order to propose a mechanism for GFS anodic behavior at this electrode.

### 3.2. Electrochemical Simultaneous Determination of PAR, PHE and GFS at PGE*

**Figure 4:**
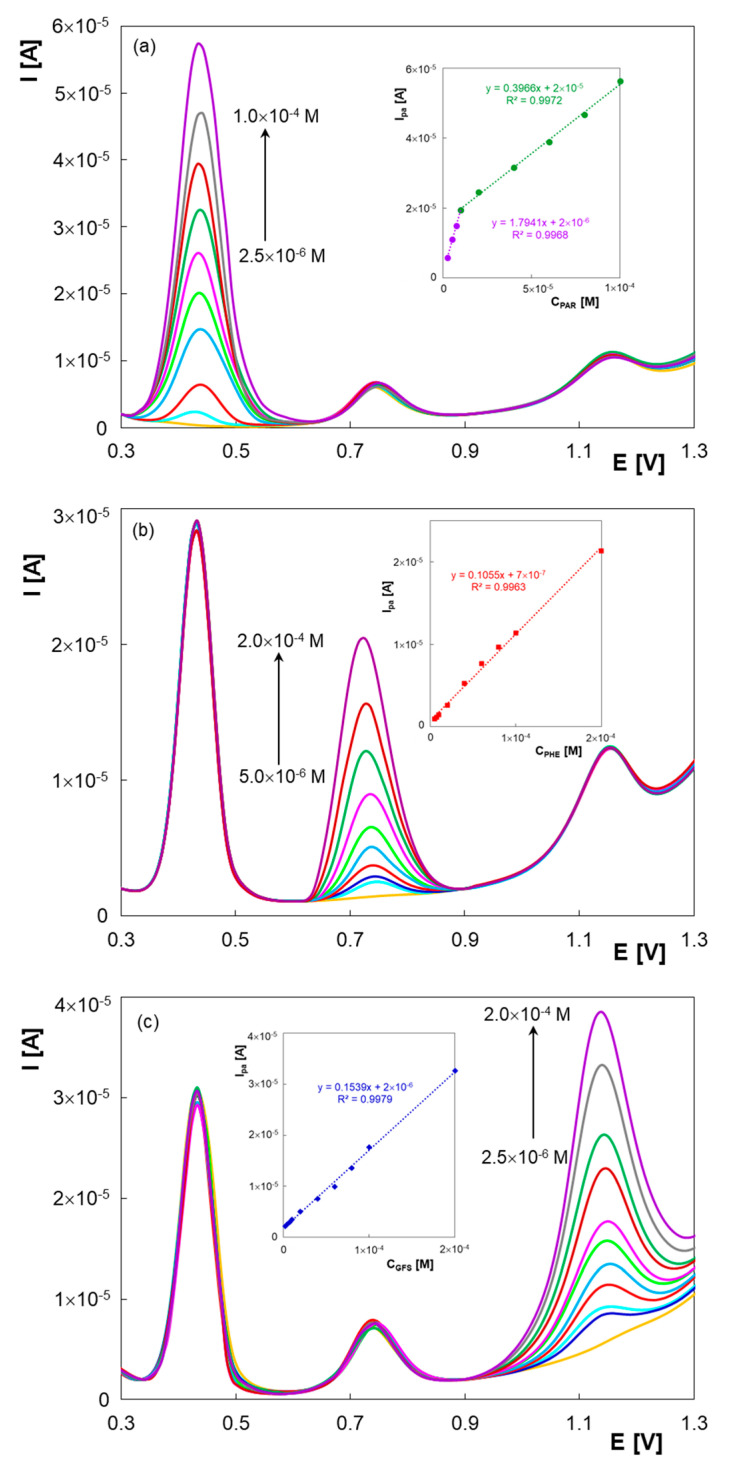
Differential pulse voltammograms of PHE, GFS (2.00 × 10^−5^ M each) and different PAR concentrations (**a**), PAR, GFS (2.00 × 10^−5^ M each) and different PHE concentrations (**b**) and PAR, PHE (2.00 × 10^−5^ M each) and different GFS concentrations (**c**) at PGE* in BR buffer solutions pH 6.00.

Taking into consideration the previously optimized experimental parameters (BR buffer solution pH 6.00, electrochemical activated HB graphite leads), DPV was employed to investigate the features of PGE* in terms of linear ranges and detection and quantifications limits for PAR, PHE and GFS. Figure 4 displays the differential pulse voltammograms recorded when all three compounds were present in the same solution. Thus, the concentrations of two compounds were kept constant while the concentration of the third was varied. In these conditions, the peak current at 0.43 V proportionally increased with PAR concentration (Figure 4a) from 2.50 × 10^−^^6^ M to 1.00 × 10^−^^5^ M, according to the equation I_pa1_PAR_ [A] = 2.3943 × C_PAR_ [M]—4.44 × 10^−^^6^ (R^2^ = 0.9974) and between 1.00 × 10^−^^5^ M and 1.00 × 10^−^^4^ M following the relationship I_pa2_PAR_ [A] = 0.3966 × C_PAR_ [M] + 1.57 × 10^−^^5^ (R^2^ = 0.9972). It can be observed that the slope of the calibration line for the second linearity range was smaller, which demonstrates a lower sensitivity at higher concentrations. This may be due to the formation of a thicker layer of analyte on the electrode surface leading to the hindering of the analyte diffusion towards the electroactive centers of the sensor or to electrode surface coverage with reaction products [42]. 

The linear ranges for PHE and GFS were 5.00 × 10^−^^6^ M–2.00 × 10^−^^4^ M (I_pa_PHE_ [A] = 0.1055 × C_PHE_ [M] + 6.97 × 10^−^^7^, R^2^ = 0.9963) and 2.50 × 10^−^^6^ M–2.00 × 10^−^^4^ M (I_pa_GFS_ [A] = 0.1539 × C_GFS_ [M] + 1.67 × 10^−^^6^, R^2^ = 0.9979), respectively (Figure 4b,c). 

The limits of detection (LOD) and quantification (LOQ) of PAR, PHE and GFS were calculated from each linear calibration curve as 3.3 and 10 times, respectively, the ratio between intercept standard deviation and slope of the regression line of the target analyte. LOD and LOQ values were: 8.12 × 10^−^^7^ M and 2.46 × 10^−^^6^ M for PAR, 1.80 × 10^−^^6^ M and 5.46 × 10^−^^6^ M for PHE and 8.29 × 10^−^^7^ M and 2.51 × 10^−^^6^ M for GFS. 

Accuracy, repeatability and intermediate precision of the voltammetric quantification method of PAR, PHE and GFS were assessed at three different concentration levels (lower, middle and higher) within the linear ranges of the analytes over a single day (intra-day assay, n = 3) and for three consecutive days (inter-day assay). Accuracy was evaluated by recovery from standard solutions and expressed as relative error (r.e. % = (C_found_ − C_added_)/C_added_)), precision being estimated by calculating the percentage relative standard deviations (RSD%). The results are shown in Table 1. 

Excipients are used in the pharmaceutical formulations to improve the therapeutic action of the active ingredients so it is important to investigate their impact on the electrochemical oxidation of the selected analytes. Some possible interfering compounds such as glucose, tartaric acid, citric acid and starch on peak currents of a mixture standard solution of PAR, PHE and GFS (2.00 × 10^−5^ M each) were studied using DPV. There was a negligible interference of the excipients on the voltammetric signals of the three active ingredients (signal change < 5%), thus proving the good selectivity of the proposed method. 

Ascorbic acid (AA) and uric acid (UA), the main interfering compounds in biological samples such as human serum and urine, were also added their concentrations being ten times smaller, equal and ten times higher) to the solution containing the three active substances. AA had no interfering effect on the detection of PAR, PHE and GFS because the AA voltammetric signal appeared at 0.04 V under the selected experimental conditions. In contrast, UA (E_pa_ = 0.40 V) affected the oxidation peak of PAR, so that, using the proposed method, the PAR content of biological samples cannot be evaluated with certainty.

### 3.3. Analytical Application 

To evaluate the viability of the proposed method for simultaneous determination of PAR, PHE and GFS in real samples, pharmaceutical ones were considered. The standard addition method was used to quantify the analytes and to determine the percentage recoveries. The pharmaceutical samples were prepared as described in Section 2.2*. Sample analysis* and were analyzed employing the DPV developed method and a new PGE* for each measurement. 

The differential pulse voltammograms recorded for each diluted sample solution presented a very intense oxidation signal at 0.43 V, characteristic to PAR, a smaller peak at 1.14 V due to GFS oxidation and, as expected, an even smaller anodic wave at 0.74 V, assigned to PHE. The difference between the peak currents corresponding to each of the three analytes was due to the fact that the content of PAR, GFS and PHE in the pharmaceutical product was very different. It must be mentioned that there was no other peak present in the samples’ voltammograms, this fact suggesting the absence of interferences from the excipients found in the pharmaceutical formulation. 

It was ascertained that the peak currents increased linearly with the addition of the appropriate stock solution. This allowed the analytes quantification one by one, exploiting the corresponding peak, even though in the solution were present all three of them. 

In this way, the content of PAR, GFS and PHE in the pharmaceutical preparation consisting in the mixture of the three compounds was determined, considering the dilutions carried out during the samples preparation steps. The percentage recovery values were calculated. The results revealed a good concordance between the values declared by the manufacturer and those obtained experimentally by the DPV at PGE* method (Table 2).

## 4. Conclusions

The current paper details the first DPV study for PAR, GFS and PHE simultaneous quantification, these active pharmaceutical ingredients being usually combined for cold and flu treatment. The CV studies proved that PAR suffered an adsorption controlled process, while for GFS and PHE the electron transfer at the electrode surface was a diffusional one. The use of PGE* proved that the electrode surface electrochemical pretreatment had a beneficial effect on the oxidation of the analytes, allowing their sensitive and selective detection. Additionally, the electrode fouling effect due to PAR, GFS and PHE oxidation products can be avoided employing the disposable PGE*. Thus, the enhanced and well resolved anodic signals were exploited in a single scan for the development of an advantageous and practical procedure for multicomponent analysis. The new method offers favorable features, such as simplicity, rapidity, low-cost, accuracy, precision, sensitivity and selectivity.

The analytical application consisting in the determination of the analytes highlighted that the electrochemical method enabled PAR, GFS and PHE reliable and interference free quantification from pharmaceutical formulations containing their ternary mixture.

## Figures and Tables

**Table 1:** Accuracy and precision data of DPV method for PAR, PHE and GFS quantification; SD—standard deviation.

C [M]	Intra-Day			Inter-Day		
	Mean Concentration ± SD [M]	r.e.%	RSD%	Mean Concentration ± SD [M]	r.e.%	RSD%
PAR						
2.50 × 10^−6^	2.59 × 10^−6^ ± 4.49 × 10^−8^	3.45	1.74	2.57 × 10^−6^ ± 6.78 × 10^−8^	2.89	2.64
1.00 × 10^−5^	1.02 × 10^−5^ ± 2.11 × 10^−7^	2.07	2.06	1.02 × 10^−5^ ± 2.46 × 10^−7^	2.43	2.40
1.00 × 10^−4^	9.85 × 10^−5^ ± 2.08 × 10^−6^	−1.22	2.11	9.97 × 10^−5^ ± 2.18 × 10^−6^	−0.34	2.19
PHE						
5.00 × 10^−6^	5.15 × 10^−6^ ± 2.55 × 10^−7^	2.96	4.95	5.13 × 10^−6^ ± 2.70 × 10^−7^	5.27	2.66
2.00 × 10^−5^	2.07 × 10^−5^ ± 8.75 × 10^−7^	3.50	4.23	2.08 × 10^−5^ ± 9.14 × 10^−7^	4.40	3.89
2.00 × 10^−4^	2.02 × 10^−4^ ± 7.92 × 10^−6^	0.95	3.92	2.06 × 10^−4^ ± 8.60 × 10^−6^	4.18	2.78
GFS						
2.50 × 10^−6^	2.51 × 10^−6^ ± 1.09 × 10^−7^	0.34	4.35	2.51 × 10^−6^ ± 1.30 × 10^−7^	0.44	5.17
2.00 × 10^−5^	2.03 × 10^−5^ ± 6.92 × 10^−7^	1.61	3.40	2.07 × 10^−5^ ± 7.35 × 10^−7^	3.66	3.56
2.00 × 10^−4^	1.96 × 10^−4^ ± 4.71 × 10^−6^	−1.99	2.40	1.96 × 10^−4^ ± 4.92 × 10^−6^	−1.86	2.50

**Table 2:** Determination of PAR, PHE and GFS in pharmaceutical sample.

	**PAR**	**GFS**	**PHE**
Declared content (mg)	500	200	10
Determined content (mg)	509.12 ± 0.78	197.10 ± 1.21	10.60 ± 0.15
Recovery ± SD (%)	101.82 ± 3.92	98.55 ± 0.60	100.56 ± 1.50
RSD%	0.77	0.61	1.49

## Data Availability

Not applicable.
